# Evening Light-Emitting Diode Screen Exposure, Melanopsin-Mediated Circadian Disruption, and Optical Filtration for Sleep Hygiene: A Narrative Review in the Indian Context

**DOI:** 10.7759/cureus.113484

**Published:** 2026-07-27

**Authors:** Suraj A Dubey, Nandhini Elango, Lanisha Blessy Coelho, Monica Chaudhry

**Affiliations:** 1 Research and Development, Acieon Labs, Sleepaxa Private Limited, Surat, IND; 2 Optometry, Avinashilingam Institute for Home Science and Higher Education for Women, Coimbatore, IND; 3 Optometry, Institute for Technology and Management (ITM) Vocational University, Vadodara, IND; 4 Optometry, Monica Chaudhry Vision Institute (MCVI), Gurugram, IND

**Keywords:** blue light, blue-light filtering lenses, circadian rhythm, india, insomnia, iprgcs, light-emitting diode, melanopsin, melatonin suppression, sleep hygiene

## Abstract

Sleep complaints are common in India, where recent meta-analytic estimates place the prevalence of insomnia among the highest reported anywhere. Over the same period, digital screen use has risen steeply, with very high daily smartphone use. Light-emitting diode (LED) screens are rich in short-wavelength ("blue") light. That part of the spectrum drives the intrinsically photosensitive retinal ganglion cells (ipRGCs) that set the circadian clock. Evening screen use can therefore blunt melatonin and push circadian timing later. Screen use is not, however, the same as the melanopic light dose actually reaching the retina. Associations between screen time and poor sleep may also reflect a later bedtime, mental and emotional arousal, notifications, and the type of content viewed. A lens would not change any of these.

In this narrative review, we trace the melanopsin-ipRGC-suprachiasmatic nucleus pathway, ask how closely LED emission overlaps circadian photoreception, and weigh the evidence for spectacle-lens filtration as an adjunct to sleep hygiene, with a specific eye on India. Standard blue-light-filtering (BLF) lenses and longer-wavelength ("amber"/"orange") wavelength-selective lenses are held to the same evidentiary standard throughout. Systematic-review evidence rates the case that BLF lenses improve sleep as of low certainty, with findings that are indeterminate and heterogeneous rather than clearly negative. Trials of longer-wavelength lenses are small, clinically mixed, mostly unblinded, and at high risk of bias. Two pooled analyses of this literature reach different conclusions: an earlier one reported a small favorable effect on total sleep time, whereas a later analysis restricted to actigraphic outcomes from randomized crossover trials found no significant effect, with confidence intervals wide enough to indicate imprecision rather than a demonstrated absence of effect. What matters, recent work suggests, is a lens's measured melanopic filtering density rather than the color on its label, and this varies widely between products. Taken together, the evidence does not yet support recommending any spectacle-lens class as a treatment for disturbed sleep; the mechanistic case for lowering evening melanopic light is firmer than the clinical case for any particular lens. We set out why India is a priority setting and what an adequately powered, India-based trial would need to look like. Where filtration is used at all, it should sit alongside, not replace, established behavioral sleep hygiene and proper medical assessment.

## Introduction and background

The identification of intrinsically photosensitive retinal ganglion cells (ipRGCs) changed how we think about light and the body, showing that image formation is not the only thing the eye does [1]. These cells carry the photopigment melanopsin, the product of OPN4, and send their axons straight to the suprachiasmatic nucleus (SCN) of the hypothalamus by way of the retinohypothalamic tract, forming the main light input to the circadian pacemaker [2]. Of the several ipRGC subtypes now described, the M1 cells do most of the work of circadian photoentrainment [3]. Their responses favor short wavelengths: early action-spectrum experiments placed the peak of acute melatonin suppression near 464 nm [4] and 459 nm [5], and a later study showed that this peak is not fixed but drifts with exposure length, settling near 481-483 nm under prolonged, 6.5-hour light [6].

The light from modern LED-backlit displays is weighted towards exactly this short-wavelength region [7]. In the laboratory, evening exposure to bright or blue-enriched light suppresses melatonin, delays the clock, and leaves people sleeping and functioning worse the next morning [8,9]. It is on this footing that current consensus guidance argues for keeping melanopic (circadian-effective) light low in the hours before bed [10].

Three cautions frame what follows. First, time spent looking at a screen is not the same thing as the light dose reaching the retina. The biologically relevant quantity is melanopic equivalent daylight illuminance (mel-EDI), a standardized measure of how strongly a light source stimulates melanopsin, expressed in lux. It differs from ordinary photopic illuminance, which is weighted to daytime cone vision and therefore to the perception of brightness, and it is a physical measurement rather than the marketing term blue light. Two displays of identical apparent brightness can deliver very different mel-EDI, and the dose actually received also depends on spectral output, viewing distance, ambient room lighting, pupil size, exposure duration, and individual sensitivity [10]. Second, the observed association between evening screen use and poor sleep need not be photic at all: screens displace bedtime, provoke cognitive and emotional arousal, deliver notifications, and carry content of varying stimulation, and none of these pathways would be modified by a spectacle lens. Third, and following from the first two, evidence that screen use correlates with poor sleep is not evidence that filtering short-wavelength light improves it. We keep these three claims separate throughout.

India is a place where these threads may come together. The 2025 meta-analysis noted above estimated a pooled insomnia prevalence of 25.7% [11], while industry figures point to roughly 1.1 trillion smartphone hours in 2024, close to five hours per user daily [12], on the back of near-universal mobile-internet access among connected groups [13]. Those usage numbers come from market and press reporting rather than peer-reviewed epidemiology, and we treat them as background rather than clinical evidence. Peer-reviewed Indian observational data do exist and point in a consistent direction: an analysis of 16,292 adolescents and young adults drawn from the UDAYA survey found that those using a smartphone for more than two hours daily had higher odds of reporting sleep problems than non-users [14]. That is an association within self-reported cross-sectional data, not a measurement of melanopic exposure and not a test of any intervention. The lenses most often sold for eye comfort and sleep in this market are standard blue-light-filtering (BLF) designs, yet the 2023 Cochrane review judged the evidence for any sleep benefit to be of low to very low certainty [15]. Our aim here is to follow the mechanism from evening LED exposure to circadian disruption and then to ask, holding every lens class to one standard, whether spectacle filtration can be defended as an adjunct to sleep hygiene, and to be candid about what the Indian evidence does and does not yet show.

## Review

Search strategy and scope

This is a narrative review conducted in accordance with the general principles of the Scale for the Assessment of Narrative Review Articles (SANRA). It does not follow PRISMA systematic-review methodology and was not registered on PROSPERO. A structured but non-exhaustive literature search was performed in PubMed/MEDLINE, Google Scholar, and the Cochrane Library, initially between January and April 2026 and updated to 21 July 2026, supplemented by hand-searching of reference lists (Table 1; full search strings, restrictions, and record counts are given in the Appendix). Peer-reviewed human studies published in English between 2001 and 2026 were eligible. Because no peer-reviewed epidemiological source reports certain India-specific usage figures, selected government and industry or press sources were used for demographic context only and are labeled as such.

**Table 1:** Literature search strategy LED - light-emitting diode; RCT - randomized controlled trial

Source	Representative search terms	Date range	Selection focus
PubMed/MEDLINE	Melanopsin AND melatonin suppression; ipRGC AND circadian; blue-light filtering lenses AND sleep; amber OR blue-blocking glasses AND sleep RCT	2001-2026	Peer-reviewed human studies; RCTs; mechanistic studies
Cochrane Library	Blue-light filtering spectacle lenses; blue-blocking glasses sleep	2001-2026	Systematic reviews; RCTs
Google Scholar	LED screen melatonin; circadian disruption smartphone; blue-blocking glasses meta-analysis	2001-2026	Peer-reviewed studies; meta-analyses
Government/industry (context only)	India sleep prevalence; smartphone screen-time statistics; UJALA LED program	2015-2025	Demographic/exposure context; explicitly labeled grey literature

No new meta-analysis, meta-regression, or recalculation of study-level effects was undertaken. This review provides a narrative synthesis and reports the quantitative results of previously published pooled analyses as those authors reported them. Certainty statements are described as GRADE-style because the assessment follows the GRADE domains of risk of bias, inconsistency, indirectness, imprecision, and publication bias without executing a formal GRADE process with a guideline panel. The domain-by-domain reasoning behind each judgment is set out with the certainty summary later in this review.

The initial search, screening, and appraisal were performed by author SD. All authors reviewed and approved the included studies prior to initial submission. Because author SD reports commercial and intellectual-property interests in this product category, and in response to peer review, two authors (NE and LBC), neither of whom declares any commercial or intellectual-property interest, re-appraised study eligibility, risk-of-bias judgments, and certainty ratings using a structured form, working from the primary sources and without reference to the original judgments. We note the limitation that both had reviewed and approved the submitted manuscript, so this is duplicate appraisal by co-authors without a commercial interest rather than appraisal by fully external reviewers. The outcome of that re-appraisal, including points of disagreement and the amendments they produced, is reported in Appendix 1. Given the narrative design, study selection remained purposive and susceptible to selection bias, which we regard as a material limitation rather than a resolved one.

The melanopsin-ipRGC-SCN pathway 

**Figure 1:**
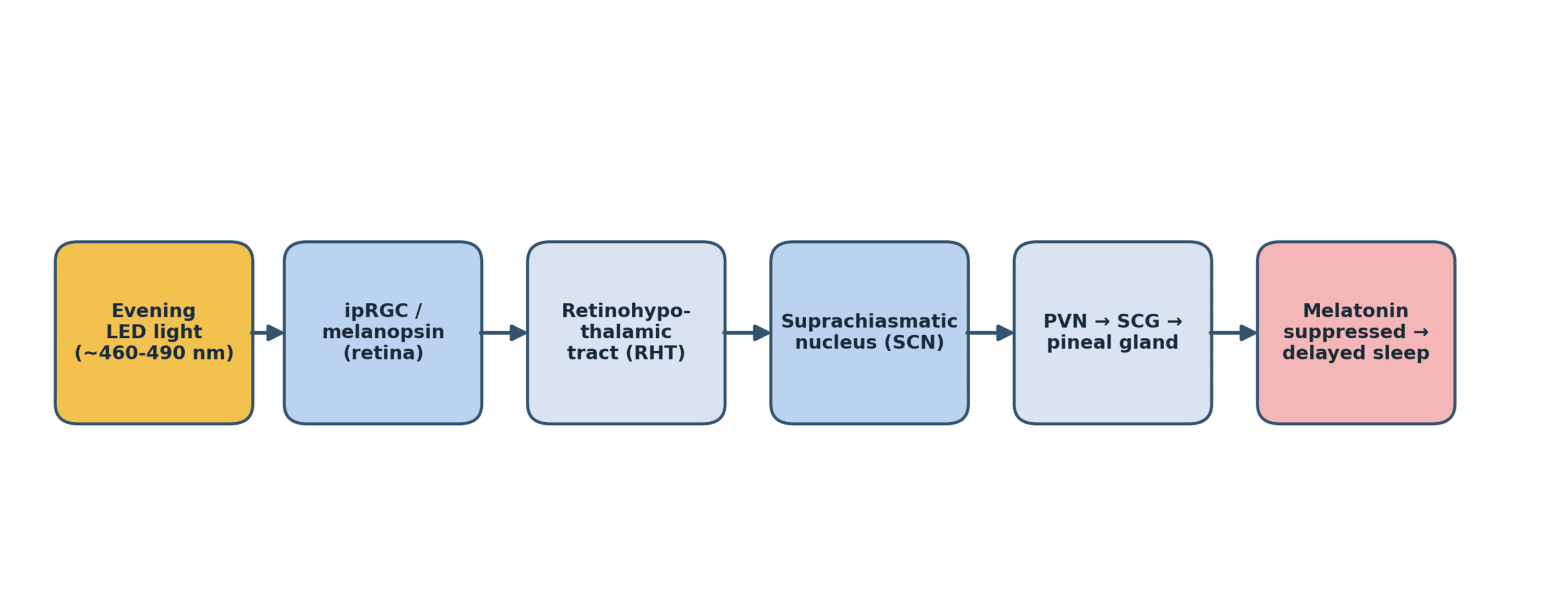
The melanopsin-ipRGC-SCN pathway mediating photic circadian regulation Evening short-wavelength light activates melanopsin-containing ipRGCs, which signal via the retinohypothalamic tract to the suprachiasmatic nucleus and, through the paraventricular nucleus, superior cervical ganglion, and pineal gland, suppress melatonin secretion, depending on both light intensity and exposure duration [1,2,16]. ipRGC - intrinsically photosensitive retinal ganglion cell; RHT - retinohypothalamic tract; SCN - suprachiasmatic nucleus; PVN - paraventricular nucleus; SCG - superior cervical ganglion

M1 ipRGCs express the highest density of melanopsin and project monosynaptically to the SCN (Figure 1) [2,3]. Photon absorption near the melanopsin sensitivity range triggers a phospholipase C-mediated cascade producing sustained depolarisation that encodes ambient irradiance to the SCN [1,2]. The SCN relays this information, via multisynaptic projections through the paraventricular nucleus and superior cervical ganglion, to the pineal gland, where melatonin is synthesized under dark conditions and suppressed by light in an irradiance- and wavelength-dependent manner [16]. Photoreceptor contributions are not fixed: cone and rod signals feed into ipRGCs, and their relative contributions vary with irradiance, exposure duration, and age, with cone input becoming more influential in older adults [6,17]. This duration and age-dependence is central to interpreting real-world evening screen exposure, which is typically prolonged rather than brief.

The circadian action spectrum

Early monochromatic-exposure studies established that acute melatonin suppression is maximally sensitive to short-wavelength light, with reported peaks of 464 nm over 90-minute exposures [4] and 459 nm over 30-minute exposures [5]. These are short-duration action-spectrum peaks and should not be equated with a single fixed photopigment maximum. Using 6.5-hour exposures in 100 participants, a later study found that the action spectra for melatonin suppression and phase resetting peaked near 481 nm and 483 nm, respectively, and that sensitivity changes dynamically with exposure duration (Table 2) [6]. The practical implication is that prolonged evening exposures, such as extended smartphone use, are likely mediated toward the ~480 nm region rather than the shorter peaks derived from brief laboratory exposures. These melatonin-suppression action spectra are downstream, system-level responses; they are correlated with, but not identical to, the melanopsin/melanopic sensitivity function, which peaks nearer 490 nm and underlies the standardized melanopic quantities defined in the international standard CIE S 026:2018.

**Table 2:** Selected human studies characterising short-wavelength sensitivity of the circadian system n - number of participants; nm - nanometres; PNAS - Proceedings of the National Academy of Sciences; NA - not applicable

Study	Year	Journal	n	Exposure	Key finding
Brainard et al. [4]	2001	J Neurosci	72	90 min	Acute melatonin-suppression action spectrum; peak ~464 nm
Thapan et al. [5]	2001	J Physiol	22	30 min	Independent action spectrum; peak ~459 nm
Gooley et al. [8]	2011	J Clin Endocrinol Metab	116	<200 lux	Room light shortened melatonin duration by ~90 min vs dim light
St Hilaire et al. [6]	2022	PNAS	100	6.5 h	Long-duration peaks ~481/483 nm; sensitivity is duration-dependent
Najjar et al. [17]	2024	J Pineal Res	NA	Varied	Photoreceptor contributions to suppression differ by age

LED screens and spectral overlap with circadian photoreception

LED-backlit displays typically use blue-pumped phosphor conversion, producing a primary emission peak near 450-460 nm and a broader phosphor-converted component at longer wavelengths [7]. The primary blue peak overlaps the rising edge of the circadian sensitivity curve and extends toward the ~480 nm region relevant to prolonged exposures [6,7]. Scientific and public-health advisory bodies have noted that LED sources emit proportionally more short-wavelength energy than the incandescent lighting they replaced [18,19]. Those assessments concern general LED illumination rather than self-luminous electronic displays; we cite them for the spectral principle alone, and they should not be read as quantifying exposure from smartphones or computer screens. The clinically relevant quantity is melanopic (circadian-effective) illuminance at the eye rather than raw display brightness; consensus guidance recommends keeping evening melanopic illuminance low, below about 10 lx melanopic equivalent daylight illuminance (mel-EDI) in the hours before sleep, and near 1 lx during sleep [10]. Typical evening device use commonly delivers melanopic light above these recommended evening levels, although absolute exposure depends strongly on display, brightness, distance, and duration. Objective evidence that this evening exposure suppresses melatonin in device users specifically comes from controlled studies: reading a light-emitting eReader before bed suppressed and delayed evening melatonin relative to a printed book [9], and varying display melanopic irradiance while holding luminance and color constant produced dose-dependent melatonin suppression [20]. We deliberately avoid quoting a single corneal-irradiance figure, because reported values vary by orders of magnitude with measurement conditions.

Sleep burden and screen exposure in India: epidemiological context

A 2025 peer-reviewed systematic review and meta-analysis of 100 Indian studies reported pooled prevalences of insomnia 25.7% (95% CI 16.3-38.0%), obstructive sleep apnoea (OSA) 37.4% (95% CI 27.8-48.2%), and restless leg syndrome 10.6% [11]. Heterogeneity was very high (I² > 98%), and the pooled OSA figure is weighted toward clinical and hospital-based samples; community-based estimates are substantially lower, so this number should be interpreted as an upper bound rather than general-population prevalence. India's young median age and rapid digital adoption (Table 3) have been proposed as amplifiers of evening light exposure. It is worth stating precisely what Indian evidence does and does not exist, because the three levels are easily conflated. Observational evidence is available: the UDAYA analysis of 16,292 adolescents and young adults reported that those using a smartphone for more than two hours daily had higher odds of self-reported sleep problems than non-users [14], and the aggregate screen-time totals cited alongside it are industry and press estimates [12,13]. What is absent is everything further along the causal chain. No Indian study has measured screen-derived melanopic exposure at the eye, and no Indian randomized trial has tested optical filtration for sleep. The "convergence" argument is therefore a hypothesis motivating research, not an established causal finding. Reduced nocturnal melatonin secretion has itself been documented as an objective feature of primary insomnia: polysomnographic studies report significantly lower night-time plasma melatonin in insomnia patients than in matched controls, with the largest reductions in those with the longest-standing complaints [21], and diminished nocturnal melatonin production despite only minor objective sleep changes [22]. This links the hormone at the center of the proposed pathway to the disorder this review concerns, although it does not by itself establish that evening screen light is the cause.

**Table 3:** Indian demographic and exposure context (sources of differing evidentiary strength; see note) Rows draw on sources of unequal strength. Only the meta-analytic prevalence figures are peer-reviewed; usage and program figures are grey literature included for context and are not used to support any clinical efficacy claim. CI - confidence interval; OSA - obstructive sleep apnoea; LED - light-emitting diode.

Parameter	Reported value	Source (evidence grade)
Pooled insomnia prevalence	25.7% (95% CI 16.3–38.0)	Peer-reviewed meta-analysis [11]
Pooled OSA prevalence	37.4% (clinical-weighted; not general population)	Peer-reviewed meta-analysis [11]
Smartphone hours, 2024	~1.1 trillion (~5 h/user/day)	Industry/press estimate [12]
Mobile-internet penetration	Near-universal in connected populations	Industry report [13]
Median age	~28 years	UN Population Division [23]
UJALA LED programme	>360 million LED bulbs distributed	Government of India (context) [24]

Optical filtration I: standard blue-light-filtering (BLF) lenses 

Blue-light-filtering lenses, as usually prescribed, remove only part of the shorter high-energy visible band. The 2023 Cochrane review of 17 randomized trials rated the evidence as low to very low certainty and did not find that these lenses improve sleep quality or ease eye strain. Its conclusions should be stated carefully: for sleep outcomes the review found the evidence indeterminate and heterogeneous rather than establishing that no effect exists, and for macular outcomes it reported an absence of trial evidence rather than evidence of absence [15]; professional bodies take the same line and do not recommend blue-blocking eyewear for routine screen work [25]. The usual mechanistic defense rests on partial overlap: many BLF products put most of their attenuation below the ~480 nm region that matters most for prolonged circadian exposure. That argument is reasonable but easy to push too far, since plenty of BLF lenses do reach into the 460-480 nm range, and a plausible mechanism is not the same as a clinical effect. Fairly read, BLF lenses at present have no solid clinical support for a sleep benefit.

Optical filtration II: longer-wavelength (amber/orange) wavelength-selective lenses

Several small studies have tested lenses that block more of the short-wavelength spectrum, and their designs are more heterogeneous than secondary accounts usually suggest (Table 4). A parallel-group randomized trial in 20 adults reported better self-reported sleep quality and mood, but its two groups differed significantly at baseline on both of those outcomes, no objective measure was recorded, and the authors note that the nominally ultraviolet-only comparator lens in fact attenuated blue light below about 460 nm [26]. A randomized placebo-controlled crossover trial in 14 adults with insomnia improved on its subjective primary outcome (p = 0.023) and on actigraphic total sleep time (p = 0.035, Cohen d = 0.65), while actigraphic sleep onset latency, sleep efficiency, and wake after sleep onset were all unchanged [27]. A counterbalanced crossover study in male adolescents found higher evening melatonin under the filter (p = 0.007), but no difference in dim light melatonin onset and no significant difference in any polysomnographic or actigraphic sleep variable; vigilance was in fact slower under the filter [28]. The two remaining commonly cited studies are weaker still: a randomised, participant-blinded crossover feasibility study in 12 students, which met its feasibility endpoints but was not powered for efficacy and found no significant sleep differences [29]; and an open-label single-arm study in nine patients with delayed sleep phase disorder, in which actigraphic sleep onset advanced by 132 minutes (p = 0.034) while the circadian marker itself, dim light melatonin onset, advanced by 78 minutes without reaching significance (p = 0.145) [30]. Read carefully, this literature offers less support for longer-wavelength lenses than its frequent summary as a set of positive trials implies.

**Table 4:** Studies of longer-wavelength (amber or orange) and blue-blocking lenses for sleep-related outcomes, with comparator, exposure schedule, effect estimates, and risk-of-bias appraisal O denotes an objective outcome measure and S a subjective one. RCT - randomized controlled trial; PIRS - Pittsburgh Insomnia Rating Scale; TST - total sleep time; SOL - sleep onset latency; SE - sleep efficiency; WASO - wake after sleep onset; DLMO - dim light melatonin onset; CI - confidence interval; n - number of participants

Study	Design and n analysed	Comparator	Exposure schedule	Reported effect	Risk of bias
Burkhart & Phelps 2009 [26]	Parallel-group RCT; n = 20 (10 per arm)	Yellow UV-blocking lens; authors note it also cut blue below ~460 nm	3 h before habitual bedtime until lights out; 2 weeks after 1 baseline week	Sleep quality S: F(1,18) = 40.08, p < 0.001; positive affect S: p = 0.001. No effect estimate or CI reported	High. Groups differed significantly at baseline on both primary outcomes; no objective measure; comparator not inert
Shechter et al. 2018 [27]	Randomised placebo-controlled crossover; n = 14 of 15	Clear lens, identical frames	2 h before bedtime until lights out; 7 nights per arm, 4-week washout	Primary outcome PIRS S: 72.6 vs 88.9, p = 0.023. Actigraphic TST O: +28.5 min, p = 0.035, d = 0.65. Actigraphic SOL, SE, WASO unchanged. No CIs reported	Moderate to high. Small n; masking effectiveness not reported; primary outcome subjective
van der Lely et al. 2015 [28]	Counterbalanced crossover, randomisation not stated; n = 11 to 13 by outcome	Clear lens of equal design; transmittance 30% vs 92%, so intensity differed as well as spectrum	18:00 until sleep onset for 1 week per arm, plus a 3 h laboratory display exposure	Evening melatonin O higher under filter: F(1,321) = 7.34, p = 0.007. DLMO O unchanged (p = 0.351). No significant PSG or actigraphic sleep difference. Vigilance O slower under filter	High. Male adolescents only; masking broken by perceived dimming; comparator differed in intensity; sleep outcomes null
Perez Algorta et al. 2018 [29]	Randomised, participant-blinded crossover feasibility study; n = 12 of 13	Blue-tinted non-blocking lens	At least 3 h before target sleep time until sleep onset; 4 nights per arm with washout	Feasibility endpoints met. Sleep comparisons O: not significant (p > 0.05). Not powered for efficacy	High for efficacy. Designed as a feasibility study; device data lost during the second period
Esaki et al. 2016 [30]	Open-label single-arm, before and after; 9 patients with delayed sleep phase disorder enrolled	None; baseline served as the comparison	21:00 until bedtime for 2 weeks within a 4-week trial	DLMO O advanced 78 min, not significant (p = 0.145). Actigraphic sleep onset O advanced 132 min (p = 0.034). No CIs reported	High. No control, no randomisation, no blinding; the circadian marker itself did not reach significance
Shechter et al. 2020 [31]	Systematic review and meta-analysis; 12 studies	Varied across included studies	Varied	TST, objective measures pooled O: Hedges g = 0.32 (95% CI 0.01 to 0.63), k = 6. Self-reported TST S: g = 0.51 (95% CI 0.18 to 0.84), k = 3	Broad eligibility admitting non-randomised and before-and-after designs; pooled effect largest for self-report
Luna-Rangel et al. 2025 [32]	Systematic review and meta-analysis; 3 randomised crossover trials, 49 adults	Control lenses used in the included trials	Varied	All actigraphic O: SOL -4.86 min (95% CI -20.23 to 10.52), p = 0.54; TST +8.75 min (95% CI -35.31 to 52.82), p = 0.70; SE -0.61% (95% CI -7.58 to 6.35), p = 0.86; WASO -1.47 min (95% CI -14.94 to 11.99), p = 0.83; I² = 0% throughout	Very small pooled sample; intervals wide enough to indicate imprecision rather than a demonstrated absence of effect

Two pooled analyses of this literature exist, and they do not agree. The earlier study, a 2020 systematic review and meta-analysis of 12 studies of interventions reducing evening short-wavelength light exposure, reported a small-to-medium favourable combined effect on total sleep time (Hedges g = 0.32, 95% CI 0.01 to 0.63, k = 6) and a larger effect for self-reported total sleep time (Hedges g = 0.51, 95% CI 0.18 to 0.84, k = 3) [31]. The later study, published in 2025, restricted eligibility to actigraphic outcomes from randomized controlled crossover trials and pooled three trials in 49 adults. It found no significant effect on sleep onset latency (mean difference -4.86 min, 95% CI -20.23 to 10.52, p = 0.54, I² = 0%), total sleep time (+8.75 min, 95% CI -35.31 to 52.82, p = 0.70, I² = 0%), sleep efficiency (-0.61%, 95% CI -7.58 to 6.35, p = 0.86, I² = 0%), or wake after sleep onset (-1.47 min, 95% CI -14.94 to 11.99, p = 0.83, I² = 0%) [32]. The 2025 analysis is therefore not the only attempt to pool this literature; it is the only one confined to objectively measured actigraphic outcomes from randomized crossover designs.

The divergence between the two is explicable rather than contradictory, and the explanation is informative. The 2020 analysis admitted a broader range of interventions and designs, including non-randomized and before-and-after studies, and pooled subjective alongside objective outcomes; the effect it detected was largest for self-report. The 2025 analysis excluded precisely those sources of optimism and was left with a very small evidence base. Read together, the two suggest that the apparent benefit of these lenses is concentrated in subjective outcomes and in weaker designs, and attenuates toward the null when analysis is restricted to randomized crossover trials with objective measurement. Equally, the 2025 confidence intervals span roughly 88 minutes for total sleep time and 31 minutes for sleep onset latency, ranges wide enough to remain compatible with a clinically meaningful benefit or a modest harm. They indicate imprecision arising from a small evidence base, not a demonstrated absence of effect, and it would be an error to cite them as proof that these lenses do not work.

Two further caveats temper any inference in favor of longer-wavelength designs specifically. First, the duration-dependent shift of the effective peak toward approximately 480 nm does not by itself establish which filter architecture is preferable, since a broad long-pass tint and a narrower band-selective design can both attenuate the melanopic region, and no trial has compared the two head to head. Second, filter architectures differ in how much photopic transmission and color rendering they sacrifice for a given melanopic reduction, but this is an optical trade-off rather than demonstrated clinical superiority. We flag explicitly that the first author holds a granted patent covering a band-selective spectral filtration design, and we therefore make no mechanistic or clinical claim favoring that architecture over a long-pass tint; the comparison remains untested. The fair summary is that the evidence for longer-wavelength lenses is early, sparse, and inconclusive in both directions.

Melanopic filtering density: why lens color is not sufficient

**Figure 2:**
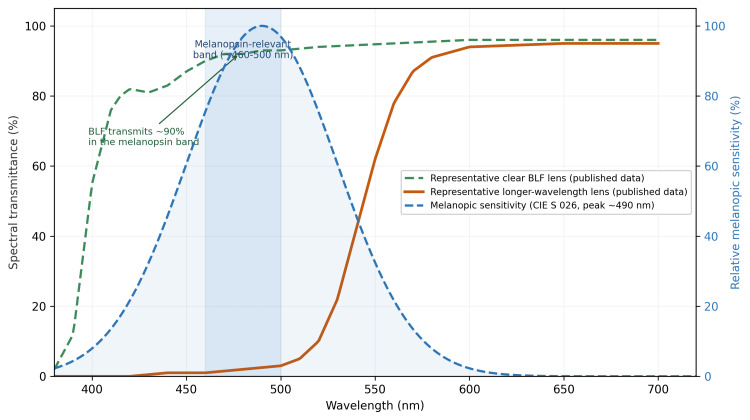
Representative lens transmittance and the melanopic sensitivity function The melanopic sensitivity function (CIE S 026; peak approximately 490 nm) [35], shown with representative spectral transmittance (%) of a clear blue-light-filtering (BLF) lens and a longer-wavelength (amber or orange) long-pass lens drawn from published spectrophotometry [36]. A clear BLF lens transmits roughly 85-95% across the melanopsin-relevant band (~460-500 nm), whereas a long-pass lens attenuates it; the biologically relevant quantity is a lens's measured melanopic filtering density, not its color. Curves are representative and illustrative, not measurements of any specific product. BLF - blue-light-filtering; CIE - International Commission on Illumination

What tends to get lost in "blue-blocking" marketing is that the property that matters biologically is a lens's measured reduction of melanopic input, not the name of its tint. A 2025 assessment of 26 commercial products introduced the melanopic daylight filtering density (mDFD) and found the spread striking: only lenses at mDFD ≥ 1 cut enough melanopic input to earn the "blue-blocking" label, and, awkwardly for the category, some products sold as "amber" managed only mDFD ≈ 0.28, while stronger orange tints reached about 1.4 [33]. A color name is therefore an unreliable guide to biological effect; what counts is the measured transmission of the particular product (Figure 2). It also means results from one high-density filter cannot simply be carried over to weaker commercial lenses. And even at near-total filtration, blue-blocking lenses lessen rather than eliminate light-driven melatonin suppression [34], which is why we avoid the phrase "virtual darkness."

Risk of bias and certainty of evidence

The question that matters in practice is a clinical one: do these lenses actually help people sleep? The evidence has to stand or fall on trial quality rather than on mechanism. The lens trials keep running into the same problems: small samples (n = 9 to 20), mixed populations (good sleepers, insomnia, delayed sleep phase disorder), the near-impossibility of blinding a visibly tinted lens, comparators that were not always optically inert, exposure periods as short as four nights per arm and rarely longer than two weeks, and outcome measures that differ from study to study. On a GRADE-style reading, the certainty is low to very low for both BLF and longer-wavelength lenses (Table 5), which leaves one conclusion that applies equally to both: the evidence is not enough to recommend either as a treatment for disturbed sleep.

**Table 5:** Certainty-of-evidence (GRADE-style) summary for spectacle-lens filtration and sleep outcomes BLF - blue-light-filtering; TST - total sleep time; SOL - sleep onset latency; RCT - randomized controlled trial; GRADE - Grading of Recommendations Assessment, Development and Evaluation

Comparison and outcome	Body of evidence	Domains downgraded	Reason for each downgrade	Certainty
Blue-light-filtering lenses vs control, sleep quality	Cochrane review, 17 randomised trials [15]	Risk of bias; inconsistency; imprecision	Trials mostly small and subject to performance bias, since a tinted lens is difficult to conceal; results inconsistent in direction and magnitude across trials; intervals wide relative to any clinically meaningful difference	Low to very low
Longer-wavelength lenses, total sleep time and sleep onset latency	Two meta-analyses and five small trials [26-32]	Risk of bias; imprecision; inconsistency	Blinding infeasible with a visibly tinted lens and comparators not always inert; the actigraphy-restricted pooled sample is only 49 adults; the two published pooled analyses disagree in direction, which is itself evidence of instability	Low to very low
Evening melanopic-light reduction, melatonin response	Controlled laboratory and mechanistic studies [4-6,8,20]	Indirectness	Laboratory effects are consistent and dose-dependent, and were reproduced with melanopic irradiance varied independently of display luminance and colour; downgraded because melatonin suppression is a physiological biomarker rather than a patient-important sleep outcome	Moderate for the biomarker; low for clinical sleep outcomes

Interpretation and clinical implications 

The mechanistic case that prolonged evening short-wavelength light can perturb circadian physiology is well supported [1-6,8-10]. The clinical case that any specific spectacle-lens filter improves sleep is not: the pooled trial evidence is null or of low certainty for both lens classes [15,32]. Applying one honest standard to both products, the defensible position is that reducing evening melanopic light exposure is biologically reasonable, but that no lens class can currently be recommended as a therapy for insomnia or any sleep disorder. Where reduction of evening light is pursued, behavioral measures (dimming, warmer display settings, reduced late-evening screen time, and a dark sleep environment) are supported by consensus guidance [10], and any optical filter selected should be characterized by measured melanopic filtering density rather than color [33]. Optical filtration should be framed as, at most, an adjunct that complements, not replaces, established behavioral sleep hygiene, and patients with persistent insomnia or suspected sleep disorders should undergo evaluation by qualified clinicians.

Why India is a priority setting, and what remains unknown

India combines a large, young, rapidly digitizing population with a high measured burden of insomnia [11], which makes it a reasonable priority for circadian-health research. However, the "Indian context" of this review is a motivation for study, not a body of Indian efficacy data: to our knowledge, no Indian trial has evaluated evening melanopic-light reduction or lens filtration for sleep, and the exposure statistics available are industry/press figures rather than measured melanopic dosimetry [12,13]. We therefore frame India as a setting where the relevant trials should be conducted, and we avoid extrapolating Western trial findings already weak to Indian clinical practice.

A research agenda

Priorities that would materially advance this field, several of them well suited to Indian populations, include the following. First, adequately powered, sham-controlled randomized trials of evening melanopic-light reduction, both behavioral and optical, using standardized outcomes (sleep onset latency, total sleep time, and sleep efficiency measured by actigraphy or polysomnography) and pre-registered protocols. Second, characterization of products by measured melanopic filtering density (mDFD), so that interventions are defined by spectral performance rather than lens color and can be reproduced across studies. Third, calibrated spectroradiometric surveys of real-world evening light and display exposure across urban, semi-urban, and rural Indian settings to quantify the melanopic dose. Fourth, comparative-effectiveness studies of optical filtration against device-based software (reduced-blue or night modes) and of combined approaches. Fifth, longitudinal studies assessing the durability of any benefit and effects in higher-risk groups such as adolescents, shift workers, and older adults. Such trials are well suited to Indian populations and should use independent investigators, blinded outcome assessment, and pre-registration to manage conflicts of interest. A further methodological priority is exposure measurement itself. Validated instruments exist for adjacent constructs, such as the short-form Smartphone Addiction Scale for problematic smartphone use [37], but to our knowledge no single validated questionnaire simultaneously captures the three parameters most relevant here, namely the average duration of evening screen exposure, its timing relative to habitual sleep, and any associated adverse effects. Indian studies would benefit either from combining existing instruments with objective dosimetry or from developing and validating a purpose-built exposure questionnaire.

Strengths and limitations

Strengths of this review include integration across photobiology, circadian neuroscience, and sleep medicine; use of the most recent duration-dependent action-spectrum data [6]; use of independent published spectrophotometry to illustrate the spectral basis of filtration (Figure 2); and, importantly, application of a single evidentiary standard to both competing lens classes, with explicit risk-of-bias and certainty appraisal. Limitations are material, and we state them plainly. The search was structured but non-exhaustive, and study selection was purposive rather than protocol-driven, so this review cannot be reproduced in the sense a systematic review can; the Appendix reports the search strings, restrictions, and record counts so that a reader can at least retrace what was done. Certainty judgments follow GRADE domains but were not produced by a formal GRADE process. Further limitations include the narrative design, with attendant selection bias; reliance on grey-literature sources for Indian exposure statistics (used for context only); the absence of any Indian clinical data on the intervention; and the authors' commercial and intellectual-property interest, disclosed in full. To mitigate interpretive bias, conclusions were deliberately constrained to what the trial evidence supports, the null meta-analytic result is presented explicitly, and no product or brand is recommended.

## Conclusions

Prolonged evening light in the short-wavelength range can suppress melatonin and unsettle circadian timing through the melanopsin-ipRGC-SCN pathway, and a young, heavily connected Indian population already carrying a sizeable insomnia burden is a sensible place to study it. Yet once standard blue-light-filtering lenses and longer-wavelength (amber/orange) lenses are held to the same clinical test, neither can currently be recommended as a treatment for disturbed sleep: the Cochrane evidence for BLF lenses is of low certainty, and the two meta-analyses of longer-wavelength lenses disagree, the earlier finding a small favourable effect on total sleep time and the later, restricted to actigraphic outcomes from randomised crossover trials, finding no significant effect with intervals too wide to exclude one. Where filtering does happen, it follows a lens's measured melanopic density rather than its color, and even a high measured filtering density says nothing on its own about clinical benefit. Cutting evening melanopic light remains a reasonable part of sleep hygiene, but it is best pursued mainly through behavior and treated as an adjunct to, not a replacement for, sound sleep habits and medical care. Adequately powered, sham-controlled trials, ideally run in Indian populations and specifying the intervention by spectral performance rather than name, will be needed before any spectacle-lens filtration earns a clinical recommendation.

## Appendices

Appendix 1: Search strategy, restrictions, and record flow

Databases searched were PubMed/MEDLINE, the Cochrane Library, and Google Scholar. The initial search ran between January and April 2026 and was updated on 21 July 2026; the results reported here reflect the updated search. Reference lists of retrieved articles and reviews were hand-searched.

Representative PubMed search strings were: (melanopsin[tiab] OR OPN4[tiab] OR ipRGC*[tiab]) AND (melatonin[tiab] OR circadian[tiab]); (blue-light filtering[tiab] OR blue-blocking[tiab] OR amber len*[tiab] OR short-wavelength[tiab]) AND (sleep[tiab] OR insomnia[tiab] OR melatonin[tiab]); (light-emitting diode[tiab] OR LED screen[tiab] OR smartphone[tiab] OR display[tiab]) AND (melatonin suppression[tiab] OR circadian phase[tiab]); and (India[tiab] OR Indian[tiab]) AND (sleep[tiab] OR insomnia[tiab] OR screen time[tiab]). Cochrane Library and Google Scholar were searched with the equivalent free-text terms, without field tags.

Restrictions applied were: human studies; English language; published 2001 to 2026, the lower bound chosen because the two foundational action-spectrum papers were published in 2001; and no restriction on study design, since mechanistic, observational, and interventional evidence are all discussed. Preprints were excluded. Sources of demographic and market context that are not peer-reviewed were admitted only for background and are labelled as grey literature wherever cited.

Record flow: The updated search was executed on 21 July 2026 and returned the following yields. In PubMed/MEDLINE: 800 records for the melanopsin and circadian string, 241 for the optical filtration and sleep string, 54 for the display light and melatonin string, and 1,945 for the India and sleep string, giving 3,040 records across the four PubMed strings. In the Cochrane Library, searching Title, Abstract, and Keyword: 13 records for blue-light filtering spectacle lenses, comprising 1 Cochrane review, 11 trials, and 1 clinical answer; and 31 trial records for blue-blocking glasses sleep, giving 44 Cochrane records. The two databases therefore yielded 3,084 records before deduplication. Google Scholar was used for supplementary hand-searching and for locating full texts; because its result counts are unstable and not reliably enumerable, no count is reported for it. The India string was deliberately broad and returned a large volume of material unrelated to melanopic exposure or optical filtration, which was removed at title and abstract screening. Duplicates were removed by digital object identifier and title matching, and titles and abstracts were screened before full-text retrieval. Thirty-four sources are cited in this review. We must state one gap plainly: the number of records advancing from title and abstract screening to full-text assessment was not recorded prospectively, and we decline to reconstruct that figure after the fact. Records considered at full text were excluded for the following reasons: intervention or exposure not relevant to melanopic light or optical filtration; outcome not sleep, melatonin, or circadian phase; conference abstract or preprint without peer review; duplicate report of an already included dataset; and, in the case of several commercial white papers on lens transmittance, absence of any peer-reviewed publication or independent verification. We recognize that the absence of a full stage-by-stage count is a limitation the reviewer was right to raise. Database yields are reported above because they are verifiable by re-running the stated strings; the intermediate screening count is not reported because it was not recorded, and we prefer to say so rather than supply a figure we cannot substantiate. Any future review by this group will record screening counts prospectively at every stage. Because the search remained purposive rather than exhaustive, these yields describe what was searched and screened and should not be read as a PRISMA record flow.

Reviewer roles were as follows: SD conducted the initial search, screening, and appraisal. All authors reviewed and approved the included studies before initial submission. Following peer review, NE and LBC, neither of whom declares a commercial or intellectual-property interest, independently re-appraised study eligibility, risk-of-bias judgments, and certainty ratings using a structured form covering every included trial and every certainty judgment. The outcome was as follows. Both appraisers confirmed no competing interests and agreed that all included studies were appropriately selected, with none to add and none to remove. On risk of bias, one appraiser proposed a wording refinement, that the masking limitation of one trial be described as masking effectiveness not reported rather than as an absent masking check, which was adopted; the other appraiser initially recorded disagreement but clarified that this concerned whether the identified biases warranted excluding the studies, not the accuracy of the risk-of-bias notes themselves, and confirmed that the biases were transparently handled, so the notes were retained. One appraiser also identified a design-label discrepancy for one trial, which had already been corrected in this revision from crossover to parallel-group design. On certainty of evidence, one appraiser agreed with all three ratings, while the other suggested that the low-to-very-low ratings for the two lens classes might be considered moderate, on the grounds that some studies report favorable sleep outcomes. After discussion among all authors, the low-to-very-low ratings were retained, because they follow the GRADE assessment of the 2023 Cochrane review and because the underlying trials carry serious risk of bias, inconsistency, and imprecision; raising the rating to moderate would overstate certainty against the cited source. We report this disagreement rather than resolve it silently, and note that the appraiser without a commercial interest argued for a higher certainty than the commercially interested lead author was willing to adopt, which is the opposite of the direction a product-favorable bias would take. We record this outcome rather than only the process, because a duplicate appraisal whose result is not reported provides no assurance to the reader.

Appendix 2: Basis of the certainty judgments

Certainty statements in Table 5 are described as GRADE-style rather than GRADE. The five GRADE domains were considered explicitly for each comparison, and Table 5 names the domains downgraded and the reason for each. No formal GRADE process was run: there was no guideline panel, no separate evidence-profile software, and no formal consensus procedure, and the judgments were made by the author team rather than by an independent methodological group. Readers should therefore treat the labels as a transparent, structured appraisal rather than as a certified GRADE assessment.
